# Robert Bernasconi and the challenges of a Critical Philosophy of Race: (Un)learning to read and teach the history of moral philosophy

**DOI:** 10.1177/01914537241284546

**Published:** 2024-09-16

**Authors:** Benjamin Décarie-Daigneault

**Affiliations:** 311285 Pennsylvania State University, USA

**Keywords:** Critical Philosophy of Race, ethics, modern philosophy, moral philosophy, poststructuralism, philosophy of education, decolonial philosophy, history of slavery

## Abstract

This essay is an attempt to determine what Robert Bernasconi’s body of work in Critical Philosophy of Race can teach us about the way in which we, philosophers and professors of philosophy, ought to treat our institutional heritage. What should we make, for instance, of moral claims made by philosophers of the modern era who – tacitly or explicitly – manifested certain levels of endorsement toward the Atlantic Slave Trade? How should we comprehend the conceptual tools that we have inherited from them, knowing that those were formulated alongside justificatory claims for the enslavement of Africans – claims that we now deem undoubtably and universally immoral? I extract from Bernasconi’s writings an implicit methodology that can be broken down into three main moves: (1) a historiographical work, akin to Michel Foucault’s ‘archaeological’ method, aimed at uncovering the material conditions that allowed for the emergence of philosophical ideas of the past, (2) a dialectical work aimed at interpreting this collection of historical data through the critical lens of race, and (3) a pedagogical work aimed at transforming the practice of academic philosophy in light of the critique. I conclude that his methodological contribution culminates in an invitation to revisit and transform the past of the institution by treating the history of academic philosophy as philosophically and conceptually relevant rather than merely incidental. Such a commitment toward critically engaging the past of our institution urges us to revisit the canon in specific ways.

## Introduction

The recent publication of Robert Bernasconi’s collection of essays *Critical Philosophy of Race* (2023) calls for an assessment of the philosopher’s broader contribution to this field of study. His work from the early 2000s, on the role played by Kant in the development of the concept of race, polemically challenged us – philosophers of the present – to take a critical outlook at canonical figures of the past and founders of longstanding traditions of thought.^1^ This tendency is also strongly noticeable in his work on the debates about slavery that were going strong between the 17th and the 19th century, but which remain vastly unacknowledged in the academic field of modern ethics.^2^ Over the past three decades, Bernasconi constituted a vast body of work in the field of Critical Philosophy of Race, which he defines as ‘an attempt to put philosophy to work to assist in the fight against racism by rendering racism more visible and more intelligible’.^3^ This critical endeavor notably led him to extensively study the past of his discipline, as he pinpointed the Modern Era as the nesting site of the concept of race with which we are familiar today, and which is the correlate of the various forms of racism that are operative in our worlds in the present. Bernasconi’s restless scrutiny of philosophy’s history led the late Charles Mills to describe him as ‘someone who, unnervingly, not merely knows the primary texts, but also the unpublished writings, the draft versions, the students’ lecture notes, the laundry lists, and the state of contemporaneous discussion of all of these matters (and more) in the obscure and unfindable (for us, anyway) journals of the time’.^4^ I wish here to take seriously this slightly caricatural portrait painted by Mills, and I wish to do so by thematizing the implicit methodology behind Bernasconi’s meticulous reading of modern philosophical debates around the slave trade.

I propose to read Bernasconi’s work on slavery as a critical method aimed at *transforming the past* in a specific way. As I will show, the practical purpose of Bernasconi’s scrutiny of the past aims beyond the common platitude according to which carefully studying history is a practice capable of precluding us from repeating in the present the mistakes made in the past. My contention is that Bernasconi’s method, far from treating the past as a static entity that could be studied as an inert object, rather understands it in its ongoing relationship to the present. Moreover, as I will argue, it understands this relationship as multilateral, insofar as it generally functions not solely as an investigation on the survivance of the past in the present (how the past has shaped the present), but rather as a disruption of the ways in which the institution of academic moral philosophy actively works toward the petrification of the past (a present that keeps shaping the past). More precisely, his historiographical work conveys an underlying conception of temporality in which the present ceases to appear to us as the passive inheritor of the past. We, as academic philosophers trained in the western tradition, did not simply inherit the past of slavery as an unfortunate historical residue, as if we had just moved into a house that happened to be populated by ghosts. On the contrary, we continually summon our own poltergeists as we reiterate the same philosophical tropes over and over again, as we keep ‘rehearsing old arguments drawn from canonical texts’.^5^ This, in return, speaks to another underlying thesis of Bernasconi’s work: when thematizing the slave trade as a socio-political issue, it is crucial to pay attention to the philosophical ideas that were developed in conjunction with it. This is because there is an irreducible porosity between the world of ideas, which has its epicenter in universities, and the socio-political worlds. This renders necessary the development of a historiography that would turn its interest toward the various contentions and debates that were occurring in philosophical circles and universities over the course of the Atlantic slave trade era. In other words, it is imperative that philosophy reflects on its own history as an institution if it aspires to provide a consequent account of the history of slavery. Philosophy must understand political history in its dialectical relation of reciprocal determination with the institution of philosophy. Downplaying its own role as an institution that is fundamentally implicated within the political phenomena that it attempts to grasp can only result in philosophy’s failure: obscuring its object of study instead of bringing it to light.

In what follows, I will argue that it is precisely the active complicity of the institution of philosophy in its own haunting, its obstinate desire to crystallize the past into complacent narratives, that Bernasconi has been attempting to challenge in his critical engagement with canonical and non-canonical modern figures. I will do so by exploring three interrelated aspects of his work. (1) First, I will say a few words about his historiographical work, which consists in reconstructing narratives of the past stretching beyond the boundaries of institutional history. This is the task that corresponds to the meticulous repatriation of figures, texts, events, customs, and other contextual elements that were either forcefully silenced or conveniently left out of the philosophical canon. (2) Secondly, I will describe his work as the dialectical task of examining the relations of *reciprocal determination* between the becoming of philosophical ideas across time and the becoming of given material contexts, shaped by various economic, political, and religious interests. (3) Finally, I will argue that this whole endeavor culminates in the ethical duty of challenging the past of the discipline through education, by putting an end to the long-lasting rehearsal that confines philosophy to a state of inertia, to a past that has no future.

## The past of a discipline

### Moral philosophy and the Atlantic slave trade

‘One should not judge the past through the eyes of the present’ – this is a maxim that trained academic philosophers are very familiar with. Without this helpful mantra, it would be rather difficult to engage seriously with the history of ideas without continually stumbling upon various types of embarrassment. John Locke’s documented involvement in the slave trade, which has now been a contentious topic for more than two centuries,^6^ is an obvious example of such embarrassments.^7^ Bernasconi, whose work has, on many occasions, attempted to grasp the philosophical consequences of this somber part of Locke’s life, reminds us that this simple hermeneutic principle cannot serve as a universal ‘get out of jail free’ card. If it is true that an effort of contextualization, paired with a certain interpretational charity, is necessary in order to properly read the work of philosophers of the past, the question remains as to how the methodological terms guiding such an operation should be determined. Bernasconi’s historiographical work operates a negotiation between these two potentially unproductive tendencies: (1) unilaterally condemning behaviors and ideas of the past for their failure to conform to the moral standards of the present, and (2) being unreasonably apologetic toward problematic ideas that might not have appeared justifiable even placed back in their context. This negotiation starts by making contexts and ideas meet. This is not a simple task, as it requires a ‘broad historical knowledge of the period, including familiarity with what the philosopher in question could or should have known’.^8^

Locke is obviously not the only figure that calls for this type of confrontation. Bernasconi’s work around the modern debates about slavery and the slave trade shows how the contradiction that traverses Locke’s life and work, far from being an isolated phenomenon, can be found at work in many other moral philosophers of the time. This is a claim that becomes more apparent in Bernasconi’s recent work. In his 2018 essay *Critical Philosophy of Race and Philosophical Historiography,* he mentions how recent research have shown that many figures of the ‘Scottish Enlightenment who are most associated with criticisms of slavery’, such as Francis Hutcheson, David Hume, Adam Smith, James Beattie, John Millar, and Adam Ferguson ‘lacked engagement with the problem of resolving the very problem of slavery in British society’.^9^ This contradiction between ideas and life – between thought and practice – engenders a dissonance that is hard to overcome. Bernasconi summarizes the challenge in an article from 1992: ‘How was it possible for the upholders of a culture to overlook the fact that they were acquiescing in a practice that in retrospect is self-evidently in contradiction with the most deeply held values attested to by that culture’?^10^ There is a response to that question that appears clearly – although under multiple forms that all comport their own particular variations and specificities – throughout Bernasconi’s work. The connecting thread that almost always makes the contradiction intelligible is *racism* – or what he calls ‘proto racism’^11^ to emphasize the absence of this concept within the targeted historical context. His three essays on Locke mobilize the concept of racism to untangle the contradiction. This interpretational move goes against the attempt, made by apologetic readers of Locke, to reconcile his ideas and alleged actions. Such readings generally result in affirming that his writings ultimately convey pure liberal ideals, despite and against the evil political background that he unwillingly inherited and followed. Opposing this optimistic portrait, Bernasconi comes to much darker conclusions. His proposition is that there is no actual contradiction, in Locke’s mind, between on the one hand affirming that slavery is an untenable state of existence for the human species, and on the other participating in the African slave trade. What makes the two sides cohere with one another is a fundamental racism inherent to Locke’s thought. Slavery is a ‘vile and miserable Estate of Man’^12^ – as Locke states in his *Two Treatises* – yet a certain category of humans is somehow deserving of it.

Locke articulates this in a way that is reminiscent of the classical argument for the justification of slavery that was already at work in Sepulveda’s letters to Las Casas in 1550, during the debate about the treatment of indigenous people of America by the Spaniards.^13^ The argument is that there exists a state of barbarity, below the state of civilization,^14^ that one can reach by violating natural law. Engaging in an unjust war^15^ is the paradigmatic case in which an individual or a group can be said to belong to the category which has forfeited their natural right, that is, their right to live. In such conditions, enslavement appears as a merciful alternative on the part of the civilized, who would have been in their right to kill the wrongdoer. As Bernasconi mentions, ‘It was by acting more like an animal than like a human being that one forfeited one's right to life and so laid oneself open to slavery’.^16^ Slavery is a condition that can occur in nature, and it is in civilization that it becomes aberrant. In other words, there is, in Locke’s thought, room for a legitimate type of slavery that hinges on a certain idea of ‘who count[s] as fully human’.^17^ The ‘unconditional’ freedom of humans is therefore conditional to belonging to a certain category of humans: slavery is legitimate if one of the parties appears to be on the side of nature rather than on the side of civilized mankind – either by breaking natural law, or by simply never having been part of civil society in the first place. It is in light of this conception that Bernasconi analyzes the matter at hand, that is, the fact that Locke explicitly condoned the enslavement of Africans. Through this lens, it becomes apparent that Locke’s views implicitly convey a racist belief: that Africans are somehow situated outside of civil society, and that it is not their freedom that is described when he analyzes the natural freedom of humans. Although Bernasconi fully recognizes that ‘Locke spoke neither for nor against such an exclusion’,^18^ his philosophical system nonetheless allows for it. The possibility to exclude African people from the category of civil society can serve as a conceptual background capable of justifying – or at least of rendering non-contradictory – Locke’s involvement in the slave trade. Mills summarizes Bernasconi’s reading of Locke by saying that it consists in the unveiling of a twofold racism: on the one hand, it concerns Locke’s ‘unquestioning acceptance of the racialized institutions and social structures of his time’, and on the other, it is manifest in ‘his legitimization of the “absolutist” subordination of African slaves’.^19^

### Questioning the scholarship

The puzzle of Locke’s ‘contradiction’ is an exemplary case of the type of historiographical work that aims to unveil the ‘other side’ of those philosophical texts that philosophers inherit and study in universities. This other side is the dimension that pertains to the *actual lives that brought such texts to existence*. It is not merely a matter of testing the morality of the individual who wrote them. It is rather a way to understand philosophical ideas as emerging within complex webs of concrete practices, social positionality, political affiliations, and economic interests. In so doing, we run the risk of shattering longstanding views and established interpretations of these texts that came to be, over time, deeply rooted in the institution of academic philosophy. It is by attempting to prevent such a peril for the institution that scholars proceed to craft apologetic justifications. This is what Mann and Bernasconi observe in their assessment of the situation of the scholarship around Locke’s involvement in the slave trade. As they polemically mention, ‘some recent attempts by scholars to resolve the contradiction [are found] to be far more perplexing than the contradiction itself’.^20^ I believe that this strong statement is revelatory of an underlying conception of the history of ideas that is generally operative in Bernasconi’s work. Indeed, his attention is never strictly restricted to the lives and ideas of the philosophers that he examines. This is because his interest does not lie in retrieving the original state of philosophical ideas despite and against their transformations in history. This would entail thinking of a ‘pure state of ideality’, or a moment in which ideas would perfectly coincide with the entire expressive intention of their authors. This, in turn, would require us to envisage the philosophers that we study as minds fully transparent to themselves, capable of formulating ideas that capture in language the totality of their philosophical intuitions, beliefs, and values. Obviously, a philosopher like Bernasconi, who extensively flirted with the work of Derrida for decades, could never endorse such a view.

What is the object, then, of his historiographical work? I wish here to argue that it is the historical *becoming* of philosophical ideas that Bernasconi invests in his writing. This attitude is fundamentally incompatible with the effort to come to the rescue of canonical authors, either by sweeping some of their most problematic aspects under the rug, or by constructing elaborate systems aimed at overcoming these difficulties in order to maintain the intellectual authority of these authors over the discipline, and their active role in the unfolding of contemporary academic philosophy. Of course, it is entirely possible to defend and adopt the views of a canonical author all the while recognizing the fundamental instability of their ideas, and their inevitable subjection to historical transformations, interpretations, and reprises. This effort becomes ‘concerning’, however, when it is done *in the service of* apparatuses of power. In section two, I will say more about the reciprocal interaction between power structures and the institution of philosophy. For now, it suffices to say that there exists a tendency in academic philosophy to defend certain readings of past philosophers, and that this tendency – which influences the becoming of philosophical ideas, the intronization of certain thinkers and the rejection of others into oblivion – is an integral part of Bernasconi’s object of study. For the question that guides his investigation is not merely whether or not canonical thinkers like Hegel, Kant or Locke were racist in their time. Rather, he is interested in knowing ‘why these philosophers are still regarded as canonical in areas where they were clearly deficient compared with other thinkers who are still ignored in philosophical circles’.^21^ This is the purpose that guides his historiographical investigation and his scrutiny of these philosophers: the treatment that they received within scholarly circles over the decades and centuries is revelatory of the institution of philosophy itself, of its becoming, and of its relationships with the other structures that shape our cultural worlds.

## The culpability of an institution

### Historiography, archaeology, and genealogy

How is Bernasconi’s unveiling of Locke’s racism not a way of petrifying the past in *another way*? How is it not an attempt to establish a new *dominant interpretation* that should forever reign over Locke’s scholarship? And most importantly, if it is not any of that, to what sort of truth or validity can an interpretation of the sort pretend? What I wish to argue is that Bernasconi’s sensitivity to the unstable nature of ideas across time leads him to treat his own work as part of broader motions of change and becoming within thought. His research is therefore primarily practical, insofar as it aims to *do something* to thought. His effort to tamper with the past – to be concerned with the past of his discipline – *does something* to the discipline. It is by means of not leaving the dead alone, of not leaving the past untouched, that he eventually acquired the reputation of being somewhat of a troublemaker within the discipline: Bret Davis went as far as calling him the ‘Gadfly of Continental philosophy’. Davis coins this humorous title to capture what he believes to be the essence of Bernasconi’s work, that is the fact that ‘[he] unsettles us, challenges us to be more selfcritical, and thereby keeps us on the move toward greater wisdom and justice’.^22^

So far, examining the case of Locke has made salient five essential elements of Bernasconi’s methodology. (1) It questions the heritage of academic philosophers of the present by critically engaging canonical figures of the past. (2) Its object is not the retrieval of what would be the ‘true’ philosophical intention of these figures. (3) Its object is rather the historical becoming of their ideas, their sources of origin, the back-and-forth motion that exists between the primary texts and their interpretations in history, the readership that they reached, the political views that they served, the concrete effects that they had in the world, etc. (4) The figures themselves, their lives, works and conceptual systems, although they are of secondary interest in the research, must become objects of careful examination, as they serve as the primary material for the historical investigation. (5) The purpose of such examination is twofold: on the one hand, it serves the *theoretical purpose* of shedding light upon the historical becoming of the institution of philosophy in its relation to the world, and on the other, it serves the *practical purpose* of disrupting the foundations of the discipline by opposing the tendency of the ‘mainstream historians of philosophy’^23^ to work toward the preservation of a stable state of the canon.

Furthermore, I believe that it is possible to distinguish, within the method itself, three levels of analysis. (1) First, the historiographical task corresponds to the retrieval of factual information and fragments of memory scattered in writings. It consists in the gathering and the studying of raw materials susceptible to help broaden our knowledge of the past (e.g., personal letters, legal documents, variations within different editions of a book). It is an archivistic endeavor that, by paying attention to texts, ideas, artifacts, people, or events that were not retained in collective memory, aims to render available new dimensions of the past. For instance, the task of repatriating the facts concerning Locke’s involvement in the slave trade into the analysis of his philosophical interrogation of slavery belongs to this historiographical effort. (2) Secondly, the *archaeological* level of analysis is the description of the way in which the studied terms are *distributed*. It aims to reveal the relationships between the actors, events, discourses, systems, structures, and institutions that the historiographical moment unveiled. I use the word ‘archaeology’, which is rarely invoked by Bernasconi, to emphasize the proximity of this aspect of the method with Foucault’s own concept of archaeology. Bernasconi, more than familiar with Foucault’s work, has often drawn on the latter’s conceptual arsenal in his attempts to grasp the concept of race through ‘a critical genealogy that takes into account the material conditions and the interests that shaped it’.^24^ Although he remains generally suspicious of Foucault’s treatment of the question of race in his account of history,^25^ Bernasconi has demonstrated, throughout his many discussions of concepts such as biopower and biopolitics, an explicit indebtedness to Foucault’s thought. I wish to add here that his view of history and the history of ideas also stands in close proximity with that of Foucault. In *The Archaeology of Knowledge* (1969), archaeology is defined as the ‘intrinsic description of the monument’.^26^ In this case, the monument that is targeted by Bernasconi is academic philosophy as an institution. As we have seen while discussing his reading of Locke, the becoming of philosophical ideas across time tends to be marked by a form of interpretational sclerosis, which is established over the adoption of royal views across time. These tendencies to defend certain accepted views of past philosophers, as I described earlier, are all part of the ‘long and complex story to be told about how the canon came to take the shape it did’.^27^ By addressing the contradiction of Locke in relation to slavery, and by addressing it under the angle of ‘proto racism’, Bernasconi sheds light on an intricate network of relations, internal to the institution, that subtends it and structures its historical unfolding. This reveals how the royal narrative is part of other possible narratives that come to light only through this description of the intrinsic relations that compose the monument. Again, in the words of Foucault, ‘Recurrent redistributions reveal several pasts, several forms of connection, several hierarchies of importance, several networks of determination, several teleologies, for one and the same science, as its present undergoes change’.^28^ (3) This in turn leads to the third level of analysis: the genealogical work. This is the process that allows for scattered fragments of memory to become new dimensions of the past. The genealogical movement is what reveals the contingency of the structures and distributions that were uncovered in the archaeological moment. Genealogy shows how things came to be, and in so doing it shows how they can still become otherwise. It unveils the movement by which history was written, showing that the latter can still be rewritten. A genealogical method, because it demands to look at the monument as a whole, inevitably fragilizes the distribution of its parts. Genealogy calls for a reorganization of the past because it shows how contingent and fragile the narratives that constitute the monument are. This is a process of unmaking that calls for the creation of new relations, new connections between ideas and between events within the trajectories of becoming that compose the past. In sum, Bernasconi’s way of thinking through the past of a discipline, which shares core commonalities with Foucault’s understanding of history, opens up a *futurity of the past*: it envisages the past as that which always has yet to be made anew. Consequently, if it is true that one ought not to judge the past with the eyes of the present, it is also crucial to affirm that the past fluctuates according to the problems that the present throws at it. Recognizing this dynamic nature of the past is what allows us to engage in a critical genealogy of the ‘monument’ that is the institution of academic philosophy. This, in turn, is the condition of possibility for philosophy’s very future. As Bernasconi argues, ‘whether philosophy deserves to survive as an academic discipline depends in large part on whether it is willing to confront its past and be more expansive in future’.^29^ As I will now attempt to demonstrate, this confrontation – which is an integral part of the genealogical endeavor – is made necessary (1) by the nature of philosophy as a *discipline turned toward its past*, and (2) by the *problematic* nature of the genealogical method, that is, the fact that it works essentially by confronting its object with *problems.*

### Failures of the institution

In one of his many attempts to describe the task of philosophy, Merleau-Ponty proposes that it should be a task of ‘*hyper-reflection*’, that is, a methodical study ‘that would also take itself and the changes it introduces into the spectacle into account’.^30^ The reflective tradition that followed Kant’s ‘Copernican revolution’ had established that any inquiry of object is incomplete as long as it is not executed through a transcendental inquiry that would reflect on the subject’s participation to the object’s being. The perspective according to which the inquiry itself does something to the object, and that this change must be accounted for in reflection, is a late addition that coincides with the progressive exhaustion of the transcendental fervor and the rise of existentialism in France, over the decades that followed the reception of Heidegger’s *Being and Time*. This principle, which was formulated in many different ways after Merleau-Ponty, is constantly at work in Bernasconi’s method: the thematization of the world that we, philosophers, attempt to articulate, has concrete impacts on the world. Philosophy can no longer be the task undertaken by this neutral and abstract figure that Husserl called a ‘disinterested onlooker’^31^: it is an activity that is motivated by certain situations and certain incentives; it is an activity that shapes the world as it describes it; and this shaping generally occurs in a certain accordance with the particular implicit interests to which the philosopher responds. The discipline of philosophy, understood as this concrete practice embedded into a history and that responds to norms, values, ideals, interests, and injunctions that is aimed toward projects and infused with a certain conception of the world: this is what Bernasconi refers to when he speaks of philosophy as an *institution*.

In his review of Bernasconi’s most recent collection of essays *Critical Philosophy of Race* (2023), Kevin J. Harrelson is critical of the author’s tendency to build his historical reconstruction of the concepts of race and racism almost exclusively through lens and methods inherited from the continental tradition with authors like Foucault, Sartre, and Fanon. He suggests that the critical endeavor aimed at unearthing the historical development of such concepts could benefit from the theoretical inputs of other disciplines or subdisciplines, notably by engaging in a dialogue with historians of biology.^32^ While it is undoubtable that proliferating critical angles and methodologies in the analysis of a historical phenomenon can only result in the development of richer accounts, Harrelson also claims that Bernasconi’s use of methodological tools inherited from continental thinkers can almost only be explained ‘biographically’.^33^ This claim appears suspicious to me. On the one hand, it suggests that the conceptual landscape in which Bernasconi elaborates his project is contingent on his own philosophical upbringing. This is, of course, undoubtable. But on the other hand, it also suggests that other conceptual landscapes could have been just as well suited to take on the same task. I doubt that this is the case. First, as I showed earlier, the biographical is never merely anecdotal: one’s philosophical heritage, which is contingent to their particular upbringing, crystallizes a certain configuration of the institution, which expresses a certain state of interaction between the various political, economic, and historical forces that shaped it at a specific stage of its temporal evolution. In other words, one’s philosophical heritage is one of the many specific individuated forms taken by the institution over its ongoing historical becoming. There is thus something theoretically valuable in tackling the institution *from* one’s inherited positionality within it, as it allows for a privileged outlook over a certain state of the studied object.

Secondly, if we define Bernasconi’s method as something like a theoretical praxis, that is, a project that is not primarily aimed at uncovering the truth of the concept of race, but that rather attempts to perturb its becoming *within* the institution, the value of a critique operated from the inside of continental philosophy becomes evident. In fact, thinkers of this multi-facetted tradition, especially phenomenologists, have largely thematized this encroachment of theory and praxis, or the way in which certain theoretical projects can be led with the aim of yielding concrete practical outcomes. Merleau-Ponty, who largely thematized the concept of institution in its phenomenological sense (as *Stiftung*, i.e., the deposition of a meaning into the world, its publicity, its availability to other subjects, its capacity to interact with other meanings, to change, etc.),^34^ approaches the problem of institution of knowledge (or what Foucault would call the ‘monument’ of philosophy), as the ‘relation of one institutional world to another, relation of a philosopher to the societies on which he reflects – of the individual to the institution’.^35^ Philosophers strive to institute meaning by responding to previously instituted meaning, according to instituted rules and customs. Thinking about philosophy as an institution that has a historical becoming requires to pay attention to these relations and their distributions, all the while taking into account that this very exercise is itself embedded and conditioned by a certain positionality within these relations, and that it ‘introduces changes into the spectacle’ as it goes on. Bernasconi has proceeded, in his attempt to map these relational networks, to describe various levels of implication, or spheres of interaction that traverse and shape philosophical ideas across time, all the while being reciprocally shaped by these same philosophical ideas. In the introduction to the 2023 book, he discusses the now publicly known concept of systemic racism by taking the example of police abuse. He shows how cases of police brutality can be read and analyzed ‘on an individual, an institutional, and a structural level’.^36^ Individually speaking, it is possible to analyze the psychological genesis of this white police officer’s racism, which led him to act aggressively in this particular situation. While analyzing police forces as an *institution*, other factors become apparent, such as the fact that ‘some police forces lack diversity’, that police officers are not trained enough, in the United States, in techniques, of de-escalation,^37^ etc. An investigation on the *structural* factors at play in police violence, for its part, would reveal the unequal allocation of funding across police forces, the unequal ‘distribution of educational resources across the United States’,^38^ etc. According to Bernasconi, however, the task of analysis would be incomplete without another level of investigation that would target all of the aforementioned levels in their reciprocal interactions, in their forming of a *system*: ‘[these cases] can also be investigated on a systemic level in which case one is looking for what produces them and what guides the response, or lack of response, to them’.^39^

This calls for a method capable of understanding patterns of distribution not only across levels (individual and structural), or across geographical spaces (here and there), but also across *time*: ‘one has to look beyond the study of the present to recognize the genesis of the societal structures operating in any given society. The forces at work, both material and ideological, are most visible in the transformations that take place’.^40^ In other words, a systemic thought, such as the one at work in the concept of systemic racism, requires the effectuation of the three levels of investigation that I described earlier. All at once, it demands (1) a set of factual-historiographical research that aim to unearth concrete displays of racial oppression within history, (2) archaeological mappings of relations between the terms at play (who, or which instance, were the actors of such events? What specific interests were at stake? Which inherited rules or customs allowed for such events to happen?), and (3) a genealogical, critical engagement with the relational networks uncovered by the investigation (what are the underlying *ideal* and *material* conditions that made these relations possible? When in time such conditions were first instituted? How have those conditions been transformed over time?). In other words, systemic thinking, which Bernasconi qualifies as a ‘synoptic view’,^41^ is inherently genealogical. Genealogy describes systems, and doing so, it relies on historiographical and archaeological moments.

Having described, through the example of police violence, the various levels of analysis that are at work in the genealogical method, it becomes easier, as we bring back our attention to the institution of philosophy, to see how such a method is well suited to reveal not only the interactions between the different components of the world that it describes, but also its own role within that world. Back to the case of Locke, a systemic view focuses neither solely on Locke the individual (his own racism), nor on his institution (the implicit rules of the philosophical discourse at that time, the economic rules governing the institution of the Atlantic slave trade in which he was involved), nor on his structural context (the cultural structure conveying racist prejudices, the legal norms enforcing such prejudices, etc.). The systemic view genealogically grasps the interaction of all those terms: ‘It was not just Locke, but virtually a whole society that, at the very time that the modem concepts of liberty and natural rights were being framed, apparently accepted the enslavement of Africans without question’.^42^ But the systemic view does not use this factual truth about the social structures of the modern era to *justify* Locke’s racism. Rather, it understands Locke’s racism as both an *expression* of his historical situation and a *condition* for this same historical situation. This is the main argument that Mann and Bernasconi develop in their 2005 essay: ‘The *Fundamental Constitutions of Carolina* and elsewhere must be understood, not as a reflection of established norms about how slaves should be treated, but as playing a role in establishing those norms’.^43^ The genealogical method that Bernasconi mobilizes is therefore inherently dialectical, insofar as it understands the genesis of ideas in their relation of reciprocal determination with the norms that govern each level of the system. In a 2017 article addressing the work of Bernasconi, Zeynep Direk observes that the unveiling of this fundamental reciprocity is one of the most crucial building blocks of the philosopher’s methodology: ‘The task is to show how customs, practices, and institutions which philosophical discourse justifies after the fact constitute [the structure and historical origin of lived experience]’.^44^ We could say, for instance, that at the individual level, philosophical ideas generate values, principles, and world views, which participate in generating them in return. At the institutional level, the same thing happens with implicit and explicit rules and norms that determine the becoming of the institution and that constitute the latter as a ‘monument’. Moreover, in obvious cases such as the *Constitution of Carolina*, philosophical ideas become the basis for norms that acquire the authority of law.

In systemic thought, the philosopher becomes lucid in regard to her insertion within an institution that is itself embedded within broader social structures. As she criticizes those structures, she makes herself increasingly responsible for her role as a part of this institution for the becoming of the institution, for the impacts that it had on the system in the past, and the impacts that it might have in the future. This is how I understand the troubling statement that Bernasconi makes in regard to police violence: ‘To fail to engage these events at the systemic level as reflections of the society at large is a form of complicity’.^45^ Applying this idea to the reflection on the multi-leveled nature of systems gives us a better understanding of how we can think of moral philosophy’s refusal to reflect on its past and on its role in shaping the power structures within society as a failure to fulfill its responsibility as a discipline. Moreover, it also shows that the institutional nature of academic philosophy calls for a responsibility of the philosopher toward her institution. The institution is more than the sum of individual philosophers that compose it, and this is why each of them is responsible for the becoming of the whole.

### The temporality of the institution

I now wish to further discuss this heightened sense of responsibility and accountability that is discernable in Bernasconi’s philosophy by asking the following question: to which extent can a philosopher of the present be responsible for the failures of past philosophers? What does it mean to take responsibility for an entire institution? In fact, philosophy, and especially moral philosophy, is in a peculiar situation in regard to its past. Many sciences, including history, have the possibility to leave their own institutional past behind as they practice their discipline. The historical object that is studied by the historian is not necessarily part of the past of history as a discipline. The raw materials that are used by the historian are not located in the past of the discipline, but rather in the past of societies, individuals, or institutions that might very well be fundamentally external to the discipline of history. This is not the case for the moral philosopher: in most cases, the raw material of her practice is *the past of the discipline itself*. If the dialogue that most sciences have with their past is implicit, insofar as it informs their current practice without being made thematic (e.g., a doctor inherits the germ theory of disease and mobilizes it in her practice without thematizing this inheritance) moral philosophy continually summons its own past – the moral theories of Aristotle, Kant, Bentham, and so on – to advance further towards its future. This means that the past cannot ‘recede’ behind the practice of the moral philosopher, who is always in an explicit dialogue with her past. Her practice consists in teaching, criticizing, overcoming, defending, commenting, and refining her understanding of the past of her institution. Thus, contrary to many academic disciplines, moral philosophy does not have the luxury of ignoring its history as the inchoate development that led to its current achievements. That such an attitude is also generally illusory – and possibly even more so – for all sciences, is a topic for another time. What is immediately salient in moral philosophy is that *it is its past.* Its development has largely been conducted as a complex work of exegesis on major moral theories. The moral philosopher engages these theories in various ways, but always works *from* them, and this makes her responsible of questioning the fervor with which she (and the institution before her) chose to engage them and not others – perhaps less well known – theories. This is a paradox that Bernasconi formulated in a panel in 2021: ‘Philosophy presents itself as a discipline that is going to instruct us an ethics. Yet we [refuse to] examine the ethics that we have been teaching, very often a continuation of the previous forms of ethics’.^46^ It is in that sense that Bernasconi comes to affirm that the past shortcomings of the institution, in some ways, travel through time. His criticism then amounts to underscoring moral philosopher’s tendency not to examine their position within the world, the power structures that they respond to, their heritage, and the potential concrete effects of their exercise. I would sum up this aspect of the critique in Merleau-Ponty’s language, by saying that Bernasconi denounces the absence of a methodical *hyper-reflection* in the practice of philosophers.

The consequence of such a conception is that philosophers must face the fact that they have a responsibility toward the past. Modern scholars today have a duty to expose the problematic dimensions of Locke’s thinking in regard to slavery if they are truly committed to their practice. The failures that we can discern retrospectively in Locke’s legacy are not confined to the past, and this is because Locke’s ideas *are their becoming.* They are not inert artifacts, insofar as they are irrevocably tethered to our current situations as thinkers. They live through our concepts and methods, insofar as they participated in shaping the institution in which we fare. As Merleau-Ponty beautifully describes in an essay initially published in 1952, ‘All those we have loved, detested, known, or simply glimpsed speak through our voice’.^47^ This, I believe, captures the ethical meaning of existing within an institution. Because academic philosophy is the becoming of its own past, it must take accountability for what it inherits. We have a duty to critically reflect upon the Locke that inevitably speaks through our voice. The inherence of ideas to their becoming is what engenders our consistently renewed responsibility to engage them critically. Again, in the words of Bernasconi, ‘they belong to the past, but they continue into the present’. This is why he adds that he is ‘concerned with the failure of philosophers today, […] to re-examine the formation of the philosophical canon and open it up to debate’.^48^

## The responsibility of a teacher

### Throwing problems at the past

In the discussion on Locke’s racism, what is at stake for Bernasconi is never solely the effort of reconstituting what happened during Locke’s lifetime and the consequences of that on Locke’s writings. What occurs in this discussion is not a historical trial of Locke as a philosopher – the issue is not to reconstitute what happened in order to gather enough information to pass a judgment; be it moral, philosophical, legal, or political. Rather, for Bernasconi, what is interesting and valuable in putting Locke in the dock is that it allows us to pay better attention to the witness stand: it allows us to listen to what has been said about Locke’s demeanors over time. The payback of that strategy is that what it ultimately reveals is far broader than the failure of Locke as an individual. What it ultimately reveals is ‘the failure of almost all canonical philosophers within the modern period to be at the forefront of the fight against slavery’,^49^ along with the continually renewed tendency of the discipline to willfully refuse to address this failure. It reveals, at the core of the institution of philosophy, a failure that keeps unfolding: ‘The canonical philosophers, who still today provide the models for how we think of moral and political philosophy, turned their backs on the sufferings of Black slaves, and many of the scholars who dedicate themselves to studying those canonical philosophers repeat the same avoidance mechanisms’.^50^ In response to this, it is now the responsibility of the inheritors of the institution to recognize this silence, to refrain from repeating it, and to invest it thematically and critically.

But there is yet another methodological condition that must come into play in order for the philosopher to be able to reveal, thematize, criticize, and correct such failures: the method must begin with a problem. As I tried to emphasize, Bernasconi’s critical genealogy, which takes systems as its object, is not the task of establishing once and for all an arche-history of the discipline. It does not consist in retrieving a true narrative hidden behind what would be revealed as a false one. Rather, it is a targeted practice of scrutiny aimed toward the uninterrupted fragilization of the dominant view of the heritage of the discipline. If it is not aimed at constituting a definitive narrative of the past that would exhaust the meaning of the history of ideas, the method that Bernasconi uses rather makes salient the fragility of the ‘official’ narrative; it reveals that what we take for the ‘truth of the tradition’ is in fact a contingent construction that can be shaped anew under a critical investigation. This brings us back to Foucault, and the distinction that he makes between a ‘total’ history and a general history. What I referred to earlier as a ‘sclerosis of the past’, or the efforts made by certain scholars to establish a dominant, unchanging narrative that would exhaust the past of the institution, is what corresponds to Foucault’s idea of a ‘total history’. General history, for its part, is genealogically and problematically established. It is the methodical organization of heterogeneous series (temporal series of events or logical series of ideas) around problematic axes. Its ability to play at all the levels at the same time – to bring together series that might initially appear unrelated to one another all the while producing coherent narratives – is guaranteed by the gravitational pull of the problem that it throws at the past. The problem of outlining new narratives despite and against the established ones, as Foucault argues, is that it requires the determination of a new distributive principle:

[It is not] trying to obtain a plurality of histories juxtaposed and independent of one another: that of the economy beside that of institutions, and beside these two those of science, religion, or literature; nor is it because it is merely trying to discover between these different histories coincidences of dates, or analogies of form and meaning. The problem that now presents itself – and which defines the task of a general history – is to determine what form of relation may be legitimately described between these different series; what vertical system they are capable of forming; what interplay of correlation and dominance exists between them; what may be the effect of shifts, different temporalities, and various rehandlings; in what distinct totalities certain elements may figure simultaneously; in short, not only what series, but also what ‘series of series’ – or, in other words, what ‘tables’ it is possible to draw up.^51^

The difficulty, for Foucault, does not lie in having to account for a multiplicity of heterogeneous trajectories within the past. The challenge rather amounts to understanding the legitimate links that can be made across these various series. The goal of such a challenge is to make these series – these networks of temporal, spatial, and symbolic relations – communicate in a *coherent* and *legitimate* ‘series of series’. I would add here that this way of organizing series in coherent narratives is what makes the system appear, and eventually what makes it possible to engage it critically. The response that Bernasconi gives to this challenge, and his attempt at taking it up, is the formulation of a *problematic* axis that would render certain historiographical elements salient to the theorizing gaze, that would archaeologically reveal the relationship between those elements, and that would have the critical-genealogical capacity to fragilize the established relationships and prepare the terrain for the creation of new ones. The problematic axis that he chose is race. Adopting it as the gravitational epicenter for a genealogical method in order to reveal how it shaped systems and was shaped by them, however, must come with a recognition of its limits. There are, of course, other problematic axes that must eventually be thrown at the past in order to arrive at always more dynamic and complex serializations within history, approaching iterations of what Foucault would call ‘general histories’. As Bernasconi mentions, ‘race is simply one lens, albeit a historically powerful one, through which to approach systems and structures. No one should need persuading that racism functions very differently for different members of a society depending on their gender, class, sexual orientation, religion, and so on’.^52^ Nonetheless, it is by confronting the history of philosophy with the problem of race that Bernasconi has been able to organize the various series that are at play within Locke’s life and work. Those include, for instance, his personal life, beliefs, and values, his economic investments and interests, his positionality within the institution of philosophy, his positionality with the political sphere, his concept of property, his concept of liberty, his clunky anthropology,^53^ etc. The serialization of those partial series occurs in response to the question of race. It makes salient new links, new relations, that would not have appeared if this particular problem had not been thrown at history.

### Summoning new ancestors

It is crucial to mention here that such a method, which has the potential to fragilize and eventually unmake solidly established narratives, is equally animated by a positive movement, that of turning the spotlight on ‘thinkers who are still ignored in philosophical circles’.^54^ Indeed, genealogically reflecting on the canon, aside from revealing systemic and institutional failures, is also for the most part a task of pinpointing the untold successes of the past. Bernasconi’s recent essay *Ottobah Cugoano’s Place in the History of Political Philosophy* is a good demonstration of this aspect of the method. As he proceeds to the historiographical reconstruction of the discussions around the abolition of slavery around the middle of the 18th century in Great Britain, he draws on Cugoano’s book *Thoughts and Sentiments on the Evil and Wicked Traffic of the Slavery and Commerce of the Human Species* (1787) to reveal aspects of the debate that were overlooked by the work of commentators over the centuries in the field of political philosophy, as though they had never existed in the first place. In addressing these aspects of the debate, Bernasconi wishes to highlight ‘Cugoano’s radicality and his importance for a rewriting of the history of political philosophy’.^55^ As he argues, if the positions of thinkers like William Paley and Thomas Gisborne – who both presented themselves as abolitionists – were mostly arguing in favor of an abolition of the slave trade, all the while presenting doubts toward the feasibility of a concrete emancipation of the enslaved, this does not necessarily mean that more radical discourses were available at that the same time. In order to show that, Bernasconi provides a reading of Cugoano’s book in which he highlights (1) Cugoano’s rejection of a gradualist view of abolition in favor of a total and immediate ‘universal emancipation’,^56^ (2) his innovative concept of collective responsibility, and (3) his idea that those who choose to ignore the evils of slavery are morally accountable and potentially deserving of punishment.^57^ According to Bernasconi, ‘Cugoano here presented an idea of responsibility that is familiar in our time: the idea that there are no innocent bystanders and that moral responsibility extends further than those actions for which we can be held directly accountable according to legal standards’.^58^

Summoning Cugoano in this context shows how there is no true anachronism in the fact of speaking of a ‘failure’ of modern thinkers when it comes to the adoption of a strong stand against slavery.^59^ This considerably undermines the overly magnanimous attitude that many commentators can have in regard to the absence of a radical anti-slavery discourse in the corpus of modern moral philosophers. It shows that positions that we would consider morally appropriate even through our eyes of the present were, in fact, accessible and discussed in intellectual spheres during those times. In so doing, it necessarily operates a reorganization of our understanding of the past. It reveals both the weakness of certain canonical ideas and the power of forgotten discourses. This opens up a novelty within the past. Indeed, Ottabah Cugoano is not a contemporary figure, yet he is new to the canon. It is in that sense that, as I mentioned earlier, a genealogical method aims to open a futurity of the past. As it refuses to abide by the habitual rehearsal of the canonical arguments that found and structure the institution, it opens up planes of novelty within the past, new terrains for thought within a past world that is temporally long gone, but that sticks to the present through the vessel of the institution. It is only through a genealogical conception of history, that accepts the non-fixity of ideas and the everchanging character of the past throughout its various taking ups in the present, that it becomes possible to summon what I would here to call ‘new’ ancestors. This paradoxical phrase, I believe, captures the essence of Bernasconi’s attempt at ‘decolonizing the philosophical canon’.^60^ A canon descends upon us by means of heredity. It is a being of the past. Genealogy shows us that we are not powerless in the face of our inheritance, just as much as we are infinitely responsible for what we inherit. It teaches us that the past is riddled with unexplored nooks and crannies that we have a duty to investigate, that the history of our institution is populated by ancestors that we have yet to meet.

To my knowledge, Bernasconi has not yet provided a definitive and exhaustive walk-through of his method. My proposition for an outline of his philosophical practice is the mere result of my general observations on a work that is primarily accomplished in fact, and not a report on a method fleshed out in theory. This, in turn, says something about the method itself: it is primarily aimed at creating an *effect* on the world. It studies what thought has done to the world in order to change the world by means of thinking. Many members of the profession agree that Bernasconi’s work should be conceived as a form of philosophical thinking which is, above everything else, rooted in practice. Mills refers to his work in terms of *service,* claiming that ‘For many years, Robert Bernasconi has been a leading figure in the quest to make race as a subject philosophically respectable, and force the profession to recognize its historic institutional racism’.^61^ Direk, for her part, claims that a crucial lesson that she learned from his teaching is that ‘in philosophy one should have an agenda, and this should have to do with making manifest and subverting the structure of oppression one experience in one’s own society and in the world at large’.^62^ There is a practical project that unfolds throughout Bernasconi’s writing, that of transforming the institution of academic philosophy by confronting it with the problem of racism. This project culminates and takes its definitive shape in teaching. Having identified that the problem of the discipline comes from the complacent rehearsal of theories rooted in an unexamined heritage, Bernasconi primarily tackles the task of transforming the institution in his role as a teacher. Training future generations of scholars opens up the future of the discipline; teaching a critically revised canon to them is what breaks history free from its institutional idleness. It is what shapes the institution anew and opens up new futures for the past.

## Notes

1. Cf., ‘Kant as an Unfamiliar Source of Racism’, in *Philosophers on Race*, ed. T. Lott and J. Ward, Oxford, Blackwell, 2002, pp. 145–66., Cf., ‘“Will the Real Kant Please Stand Up?” The Challenge of Enlightenment Racism to the Study of the History of Philosophy’, *Radical Philosophy*, 117, 2003, pp. 13–22., Cf., ‘Kant and Blumenbach’s Polyps: A Neglected Chapter in the History of the Concept of Race’, in *The German Invention of Race*, ed. Sara Eigen and Mark Larrimore, Albany, SUNY Press, 2006, pp. 73–90.

2. Bernasconi, Robert. ‘Facing up to the Eurocentrism and Racism of Academic Philosophy in the West: A Response to Davis, Direk, and Mills’. Comparative and Continental Philosophy 9, no. 2, 2017, p. 160.

3. Bernasconi, Robert, ‘Race, Philosophy, and Political: Dialogue with Owen Williams, Sharon Achinstein, Charles W. Mill, Jennifer L. Morgan, Robert Bernasconi’, *Critical Race Conversations,* (Discussion transcript), Folger Library Institute, June 21, 2021, p. 8.

4. Mills, Charles W. ‘An Englishman Abroad: Robert Bernasconi’s Work on Race’. *Comparative and Continental Philosophy* 9, no. 2, 2017, 142.

5. Bernasconi, Robert, ‘Critical Philosophy of Race and Philosophical Historiography’, in Taylor, Paul C., ed. *The Routledge Companion to Philosophy of Race*, New York, Routledge, 2018, p. 9.

6. Bernasconi highlights that this discussion started shortly after the War of Independence: ‘Already in 1807 John Towill Rutt issued a defense of Locke against the charge of supporting slavery. […] Since Rutt, scholars have been increasingly inventive in their efforts to resolve the contradiction between the Locke who profited from slavery and the Locke who, as a theorist of natural rights, would be appealed to by opponents of slavery’. – Bernasconi R., Mann, A.M., ‘The Contradictions of Racism: Locke, Slavery, and the Two Treatises’, *in* Valls, Andrew, ed. *Race and Racism in Modern Philosophy*. Ithaca, N.Y: Cornell University Press, 2003, p. 90.

7. This is how Wayne Glausser describes Locke’s involvement in the slave trade in the opening line of his piece from 1990 *Three Approaches to Locke and the Slave Trade*: ‘Every modern scholar who takes him seriously has had to confront an embarrassing fact: John Locke, preeminent theorist of natural liberties, and an influential resource for abolitionist thinkers of the eighteenth and nineteenth centuries, actually participated in the slave trade’. – Glausser, Wayne. ‘Three Approaches to Locke and the Slave Trade’. *Journal of the History of Ideas*, Vol 51, No. 2, 1990, p. 199.

8. Bernasconi, 2018, *Op. Cit.,* p. 5.

9. Bernasconi, 2018, *Op. Cit.,* p. 8.

10. Bernasconi, ‘Locke’s Almost Random Talk of Man: The Double Use of Words in the Natural Law Justification of Slavery’, in *Metaphysik* – *Grundlegungsprobleme heute, Perspektiven der Philosophie,* No.18, ed. Rudolph Berlinger, 1992, p. 298.

11. Cf. Bernasconi, ‘Proto Racism: Carolina in Locke’s Mind’, in *Racism and Modernity, Festschrift for Wolf D. Hund,* ed. I. Wigger and S. Ritter, Berlin, LIT Verlag, 2011, p. 68.

12. John Locke, *Two Treatises of Government*, ed. Peter Laslett, Cambridge, Cambridge University Press, 1988, p. 141. *in* Bernasconi and Mann, 2005, *Op. Cit.,* p. 90.

13. Cf. *Sepúlveda on the Spanish Invasion of the Americas*, ed. and trans. Luke Glanville, David Luperh, and Maya Feile Tomes, Oxford University Press, 2023, 448p.

14. Cf. Bernasconi 1992, *Op. Cit.,* p. 297. – ‘Locke situated slavery prior to civil society’.

15. ‘A just war needs not only just reasons for its undertaking, but also a legitimate initiator, the good intention of the one waging it, and a proper method of conducting it’. – Sepúlveda, 2023, *Op. Cit.,* p. 95. The terms that determine the lawfulness of a war are more or less similar across the literature: a war is just if it aims to punish a crime perpetrated, to retrieve a stolen good, or if it is aimed at the preservation of an individual or a group. Cf. Sepúlveda, 2023, *Op. Cit.,* pp. 97-98.

16. Bernasconi, 1992, *Op. Cit.,* p. 296.

17. *Ibid*, p. 298.

18. *Ibid*, p. 315.

19. Mills, 2017, *Op. Cit.,* p. 142.

20. Bernasconi and Mann, 2005, *Op. Cit.,* p. 90.

21. Bernasconi, 2018, *Op. Cit.,* p. 9.

22. Davis, Bret W.*,* ‘Gadfly of Continental Philosophy: On Robert Bernasconi’s Critique of Philosophical Eurocentrism’, *Comparative and Continental Philosophy,* Vol. 9, No. 2, p. 119.

23. Bernasconi, 2018, *Op. Cit.,* p. 5.

24 Bernasconi, Robert. *Critical Philosophy of Race: Essays*. Oxford University Press, 2023, p. 23.

25. Cf. Bernasconi, ‘The Policing of Race Mixing: The Place of Biopower within the History of Racisms’, *Journal of Bioethical Inquiry,* Vol.7, No.2, 2010, p. 206. – In this book chapter, Bernasconi describes Foucault’s failure to appropriately account for the history of slavery in his historical account of racism. He also manifests his reserves toward Foucault’s overemphasis on the biologization of racism as the major turning point in the history of the concept.

26. Foucault, Michel, *Archaeology of Knowledge* [1969], trans. A.M. Sheridan Smith, London, Routledge, 2002, p. 8.

27. Bernasconi, 2018, *Op. Cit.,* p. 10.

28. Foucault, 1969, *Op. Cit.,* p. 5.

29. Bernasconi, 2018, *Op. Cit.,* p. 11.

30. Merleau-Ponty, *The Visible and The Invisible,* trans. Alphonso Lingis, Evanston, Northwestern University Press, 1968, p. 38.

31. Husserl, *Cartesian Meditations* [1929], trans. Dorion Cairns, The Hague, Martinus Nijhoff, 1960, p. 35.

32. Harrelson, Kevin J., ‘Bernasconi’s Guiding Argument, Book Review of *Critical Philosophy of Race: Essays*’, *Critical Philosophy of Race,* Vol. 12, No. 2, 2024, p. 402. (forthcoming)

33.
*Idem*

34. Cf. Merleau-Ponty, *L’institution, la passivité, Notes de cours au collège de France* [1954–1955], texte établi par Dominique Darmaillacq, Claude Lefort et Stéphanie Ménassé, Belin, 2015, p. 62.

35. *Ibid,* pp. 62-63.

36. Bernasconi, 2023, *Op. Cit.,* p. 2.

37.
*Idem*

38. *Ibid,* p. 3.

39. *Ibid,* p. 3.

40. *Ibid,* p. 4.

41. *Ibid,* p. 5.

42. Bernasconi, *Op. Cit.,* 1992, p. 298.

43. Bernasconi and Mann, 2005, *Op. Cit.,* p. 90.

44. Direk, Zeynep, ‘Critical Philosophy of Race as Political Phenomenology: Questions for Robert Bernasconi’. *Comparative and Continental Philosophy*, Vol. 9, No. 2, 2017, p. 132.

45. Bernasconi, 2023, *Op. Cit.,* p. 3.

46. Bernasconi, 2021, *Op. Cit.,* p. 8.

47. Merleau-Ponty, *Signs* [1960], trans. Richard C. McCleary, Northwestern, 1964, p. 19.

48. Bernasconi, 2021, *Op. Cit.,* p. 7.

49. *Idem*.

50. Bernasconi, 2023, *Op. Cit.,* p. 124.

51. Foucault, 1969, *Op. Cit.,* p. 11.

52. Bernasconi, 2023, *Op. Cit.,* p. 5.

53. In reference to the ‘randomness’ pointed out by Bernasconi in Locke’s characterization of human beings. – Cf. Bernasconi, 1992, *Op. Cit.,* p. 315.

54. Bernasconi, 2017, *Op. Cit.,* p. 9.

55. Bernasconi, 2023, *Op. Cit.,* p. 135.

56. *Ibid,* p. 136.

57. *Ibid,* p. 137.

58. *Ibid,* p. 136.

59. This reinforces a point that Bernasconi makes in the introduction of his chapter: ‘It is not anachronistic to complain about the way eighteenth-century philosophers approached the issue of slavery’. – *Ibid,* p. 124.

60. *Ibid,* p. 140.

61. Mills, 2017, *Op. Cit.,* p. 148.

62. Direk, 2017, *Op. Cit.,* p. 131.

